# Supplementary material for: Local neuroplasticity in adult glaucomatous visual cortex
Source: Sci Rep. 2022 Dec 20;12:21981. doi: 10.1038/s41598-022-24709-1 (PMC9767937; doi:10.1038/s41598-022-24709-1)
Supplement: Supplementary file 1 — Supplementary Information. [file 41598_2022_24709_MOESM1_ESM.docx]

## **Supplementary Information**

*Contents*

1. Attentional Task Performance
2. Reduced BOLD modulation associated with the severity of glaucoma in V2 and V3
3. PRF estimates projected on the cortical surface mesh for multiple glaucoma participants
4. Differences in pRF position distribution within glaucoma vs matched control pairs
5. pRF size as function of polar angle
6. PRF size changes beyond V1
7. Analysis of individual deviations in pRF properties
8. PRF position deviation as function of contrast sensitivity and MNFL thickness

### Analysis of individual deviations in pRF properties based of the No Scotoma condition

1. Relation between Variance Explained and Contrast Sensitivity

### **Attention task performance**

| Task | Mean (%) | Standard error (%) |
| --- | --- | --- |
| LCR (glaucoma participants) | 75.4 | 0.04 |
| LCR (control participants) | 87.0 | 0.08 |
| LCR SS | 75.2 | 0.06 |
| LCR monocular | 77.7 | 0.04 |

**Table S1. Performance (average and standard error) of the attention task per condition.**

### **Reduced BOLD modulation associated with the severity of glaucoma in V2 and V3**


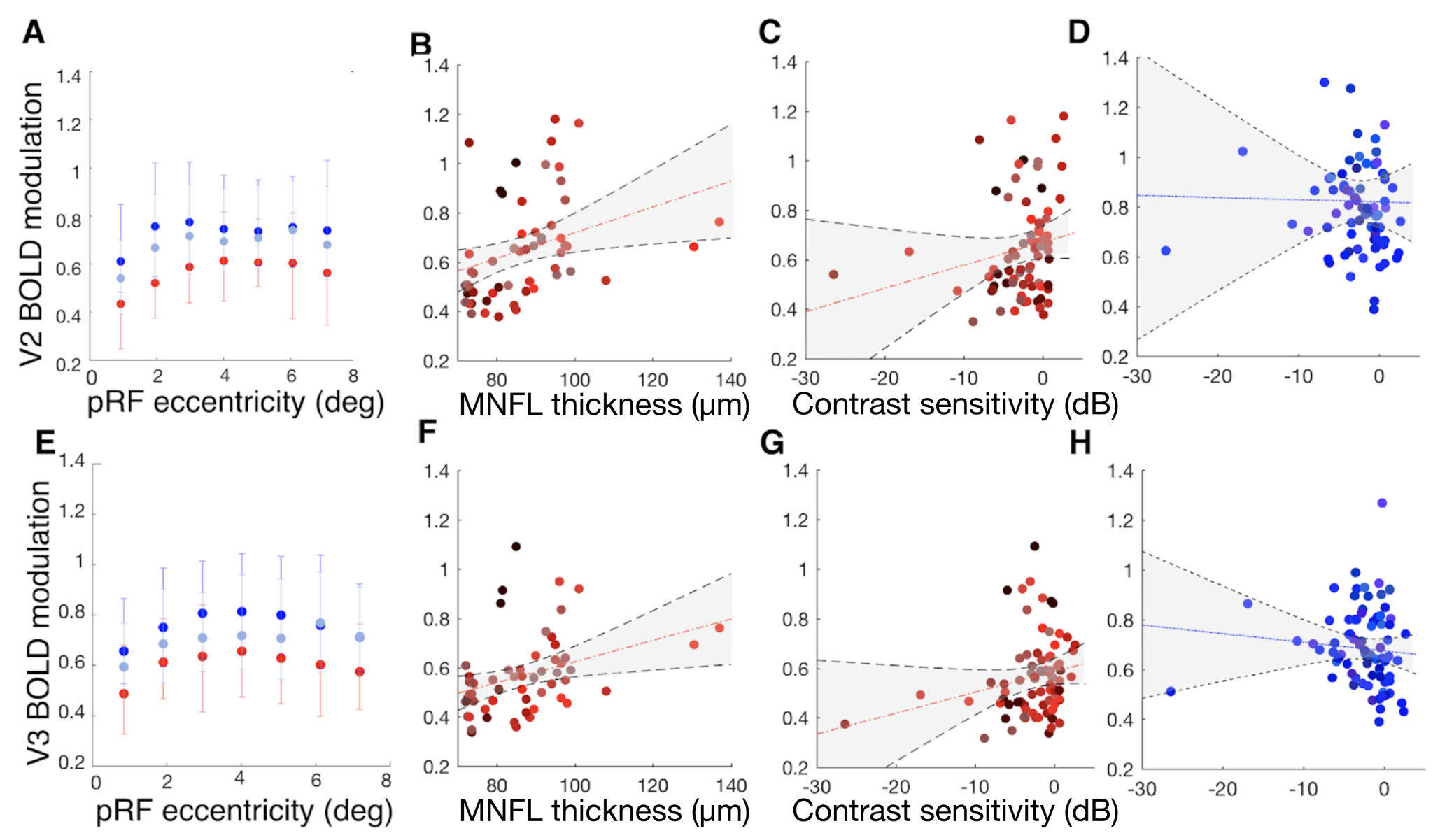


**Figure S1 - V2 and V3 BOLD modulation varies between participants with glaucoma and control participants and correlates with the severity of the disease.** A:V1 BOLD modulation as a function of eccentricity for participants with glaucoma and control participants with and without scotoma simulated, respectively red, light and dark blue. The modulation of the bold signal was binned in 1 degree bins. The error bars represent the 95% confidence interval. B: Correlation of BOLD modulation from individual quadrants with RNFL of the macula thickness. RNFL thickness was calculated by averaging the macula thickness of both eyes. Each data point is from an individual quadrant C: Correlation of the BOLD modulation from individual quadrants with visual field score (PSD) scores of both eyes combined. Each data point is from an individual quadrant. C,D,G,H: Correlation of the BOLD modulation from separate quadrants with the mean deviation (MD) of both eyes combined (the max between the MD of the two eyes) for glaucoma (red) and controls (blue). Each data point is from an individual quadrant.

### **PRF estimates projected on the cortical surface mesh for multiple glaucoma participants**

**
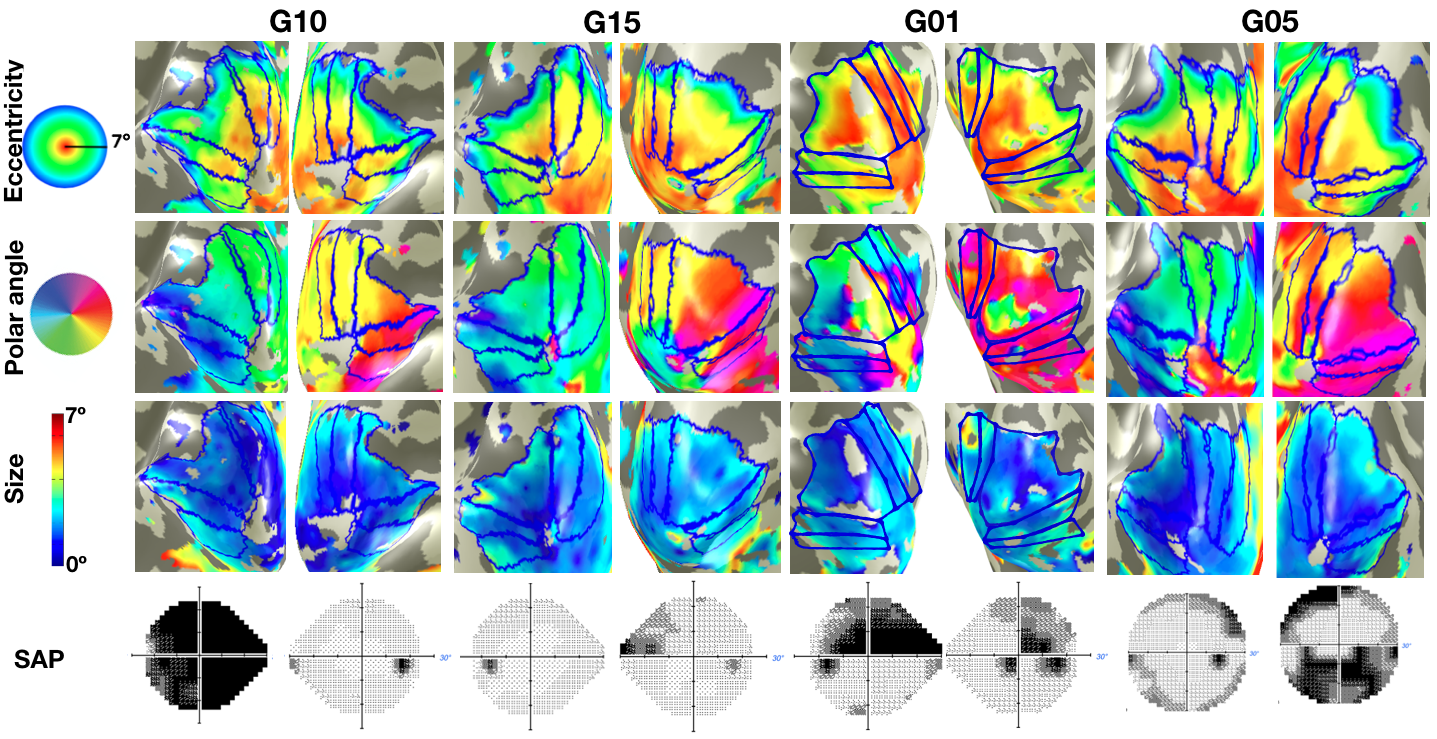
**

**Figure S2 - Preserved large-scale cortical organization in glaucoma.** Eccentricity, polar angle, and pRF size maps obtained for multiple participants with glaucoma (G02, G03, G010, G14). The maps were obtained using a explained variance threshold of 0.1 The blue lines delineate the visual areas. Bottom : VFs for the left and right eye for each of the participants with glaucoma.

### **4. Differences in pRF position distribution within glaucoma vs matched control pairs**


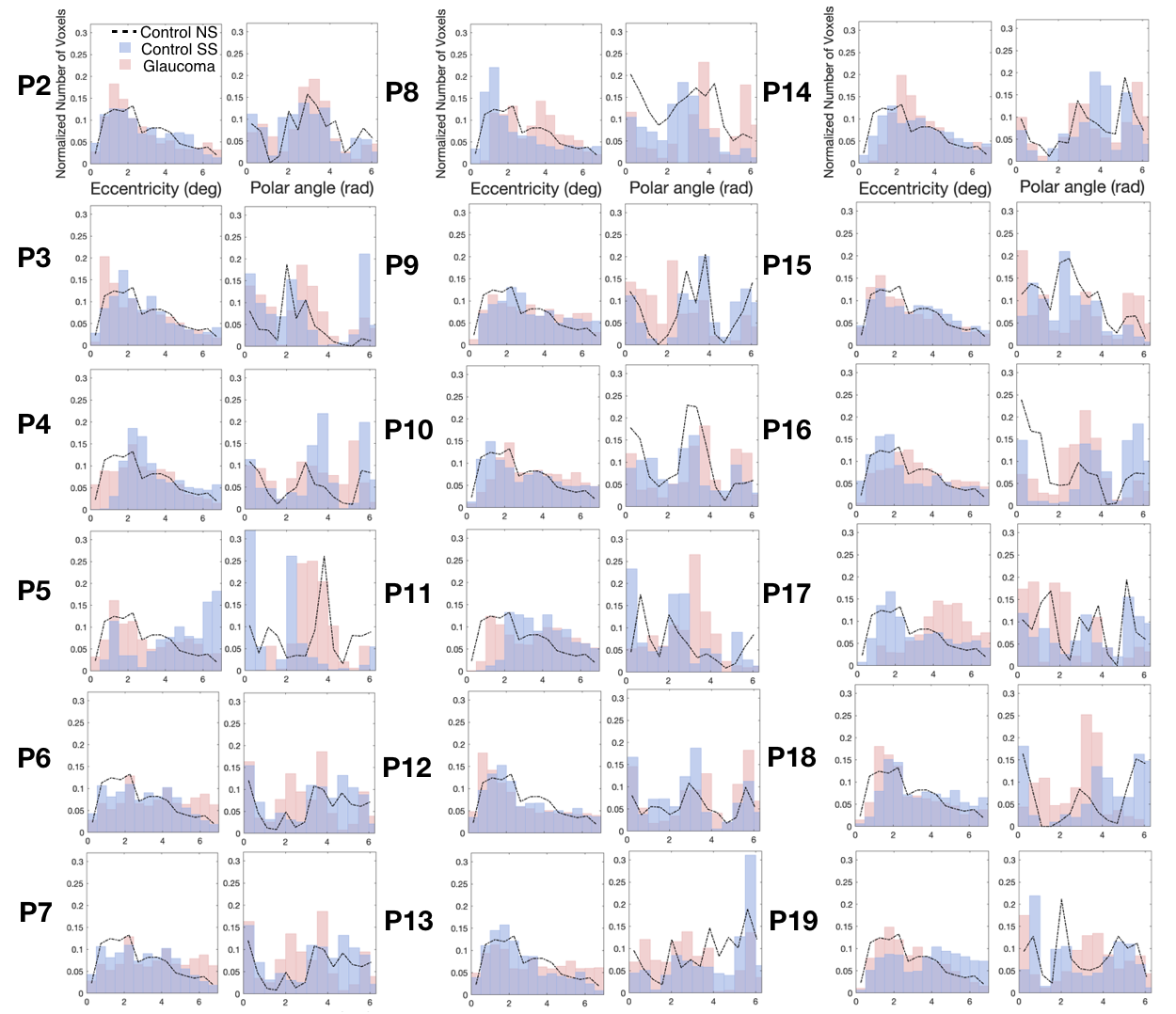


**Figure S3.**  Histogram of the normalized number of responsive voxels as a function of eccentricity and polar angle, respectively. The glaucoma participants are depicted in red and the control participant with a SS in blue. Each control participant’s results in the no simulation (NS) condition are depicted by the dashed black line.

### **5. PRF size as function of polar angle**


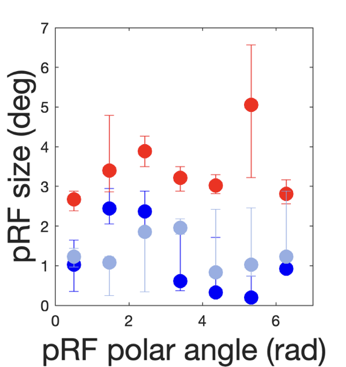


**Figure S4.** PRF size as a function of polar angle for the participant pair P01 ( glaucoma participant in red and the control participant in the conditions NS (light blue) and SS (dark blue). The error bars correspond to the 95% confidence interval.

### **6. PRF size changes beyond V1**


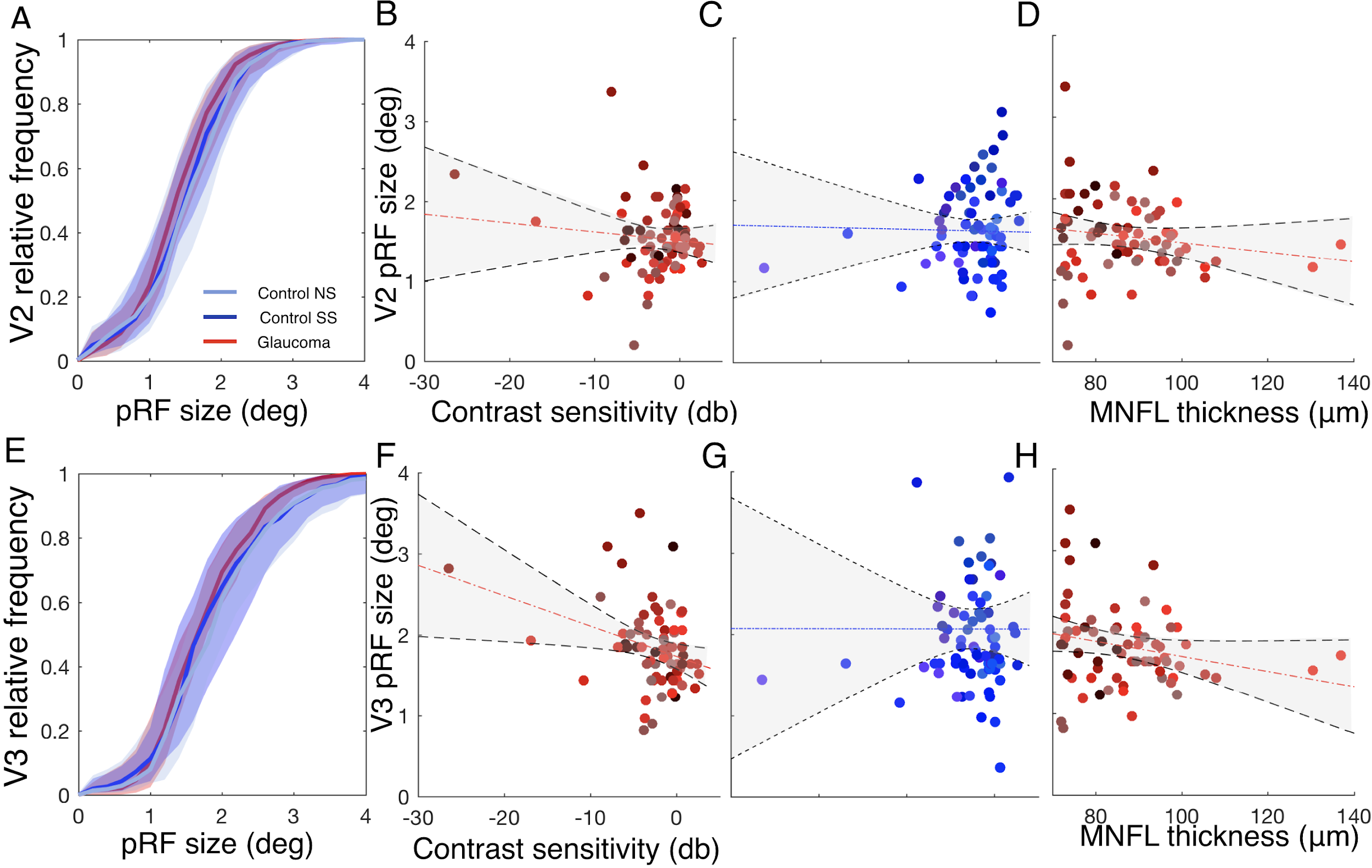


**Figure S5: Differences in pRF size for V2 and V3 between all participants with glaucoma and control participants.** Panels A, E: Cumulative distribution of the pRF size for the glaucoma participants (red) and respective control participants SS (blue) obtained for V2 and V3. The error bars represent the 10 and 90 % confidence interval between participants. Panels B, F: pRF size as a function of contrast sensitivity for participants with glaucoma. Panels C, G: pRF size as a function of contrast sensitivity for control participants. Panels D, H: pRF size as a function of retinal thickness for participants with glaucoma. The shaded gray area represents the 90 % CI between participants.

### **7. Analysis of individual deviations in pRF properties**

To establish the presence of differences in pRF properties between the participants with glaucoma and their respective control participant in the simulation condition (SS), we used the following procedure, depicted in Figures S7, S8 and S9: 1) for each participant, calculate their normalized distribution of the pRF properties (eccentricity, polar angle or size) ; 2) calculate the deviation, i.e. the difference between the normalized distributions for each participant with glaucoma and their respective control participant in the simulated condition (SS); 3) for this deviation, we determined a null distribution based on the deviations of the control participant (NS) to each of the other control participants (NS). Finally, 4) if the deviation within the specific pair (glaucoma participant - control participant (SS)) lays outside the null distribution, we consider it significant. This corresponds to applying a p-value of 0.05 (uncorrected).


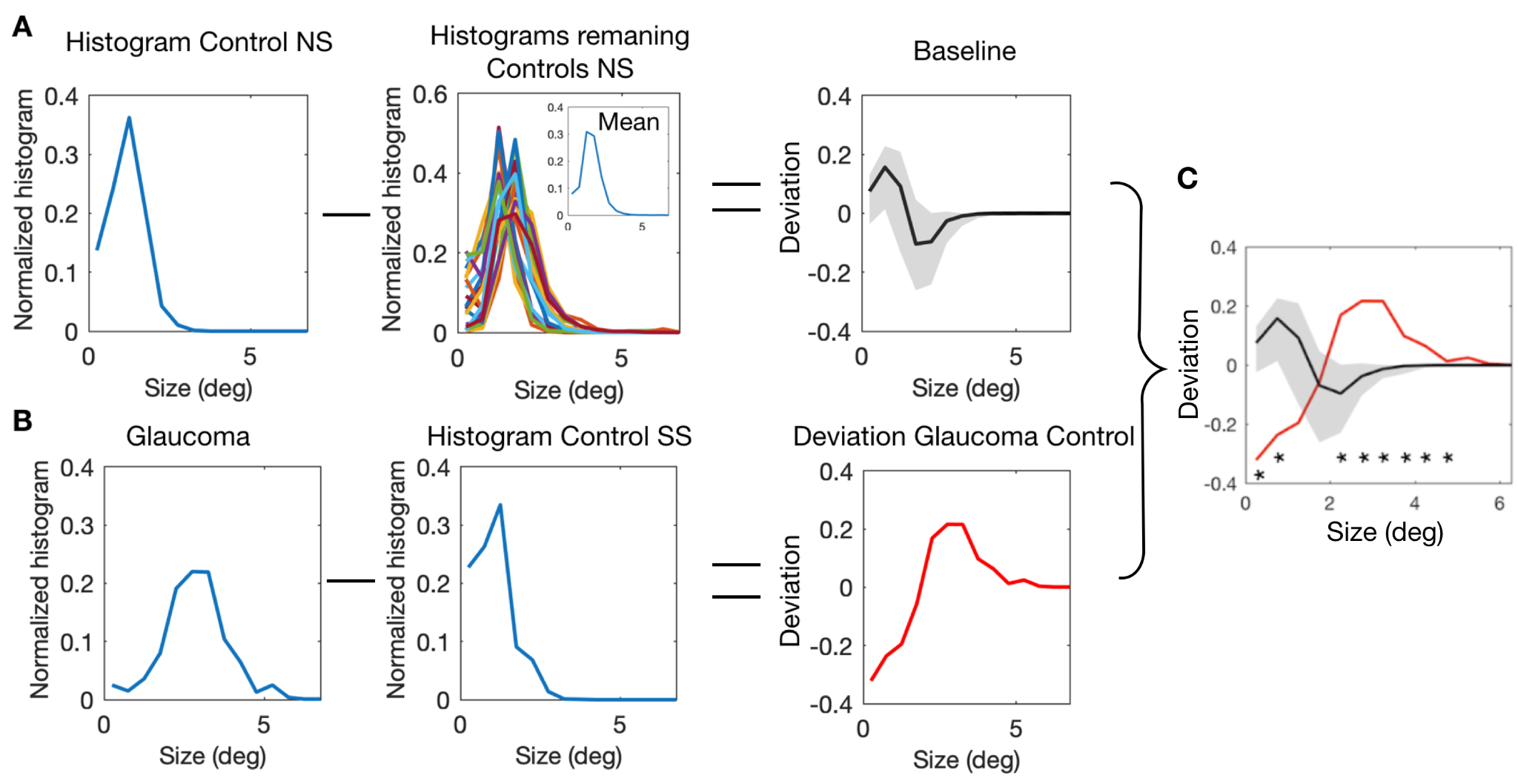


**Figure S6.** Depiction of the analysis performed to compare the pRF properties of glaucoma to those of healthy controls, an example applied to the pRF size. A: Baseline: consists of the difference between the control NS participant of interest to all the other controls NS, the black line corresponds to the median and the gray error bars to the 95% confidence interval. B: Deviation within a glaucoma - control pair (C) is obtained by subtracting the control SS normalized distribution from the normalized distribution of the glaucoma participant.

Figure S7 plots for all participant pairs the difference (glaucoma - matched control) in the proportion of pRFs with a particular size as a function of pRF size. Figures S8 and S9 also plot such deviations, but for eccentricity and polar angle, respectively. This difference with a pair can be compared to the baseline deviation, which is the average deviation between a specific control participant and the remaining control participants in the condition without simulations.


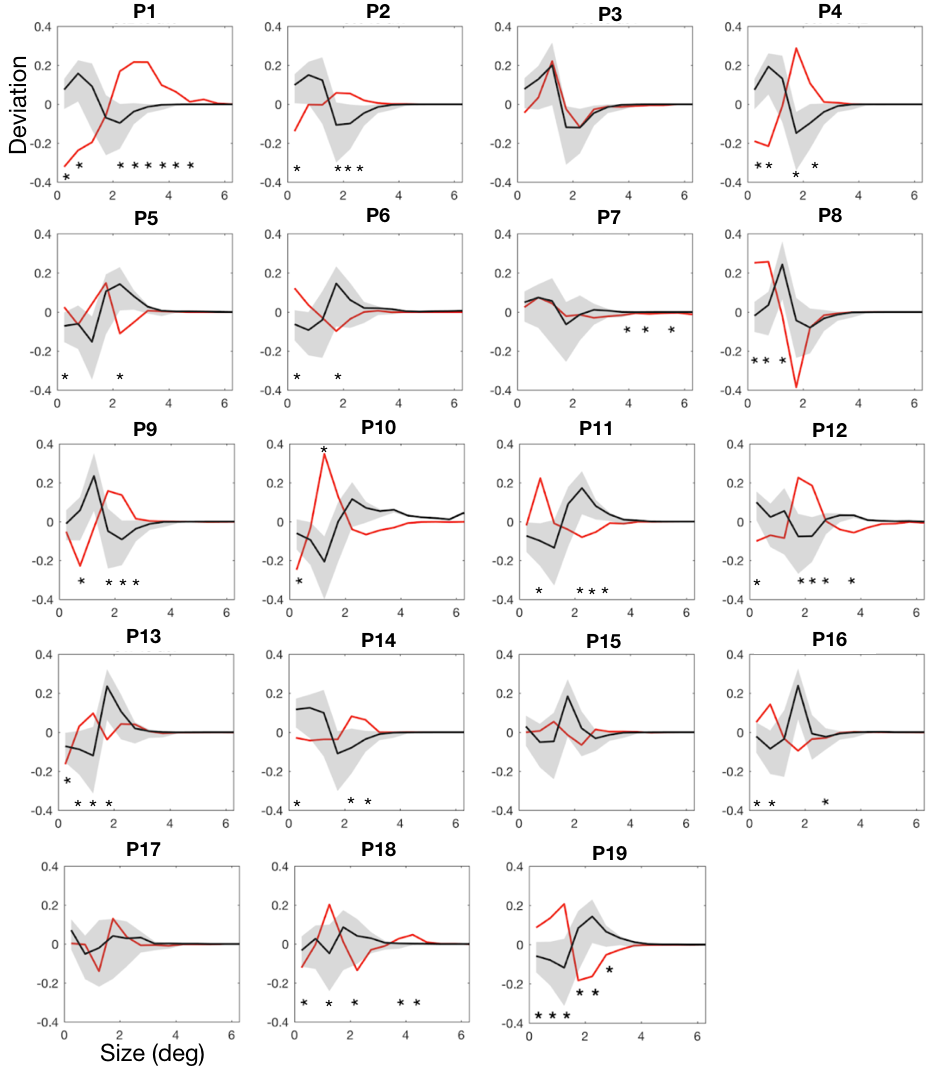


**Figure S7: V1 pRF size deviations for all participant pairs..** The red line in the plots shows, for each participant with glaucoma, the deviation in the proportion of voxels with a particular pRF size from their matched control participant with simulated scotoma (SS). The black line shows the baseline deviation for the respective control participant (NS). This was calculated as the average deviation between the matched control participant and each of the remaining control participants in the condition without simulations. The gray shaded area indicates the 95% CI. The * indicates bins in which the deviation between the participant with glaucoma and their matched control participant (SS; red line) differs significantly (p<0.05) from the deviation of this control participant (NS) from baseline.


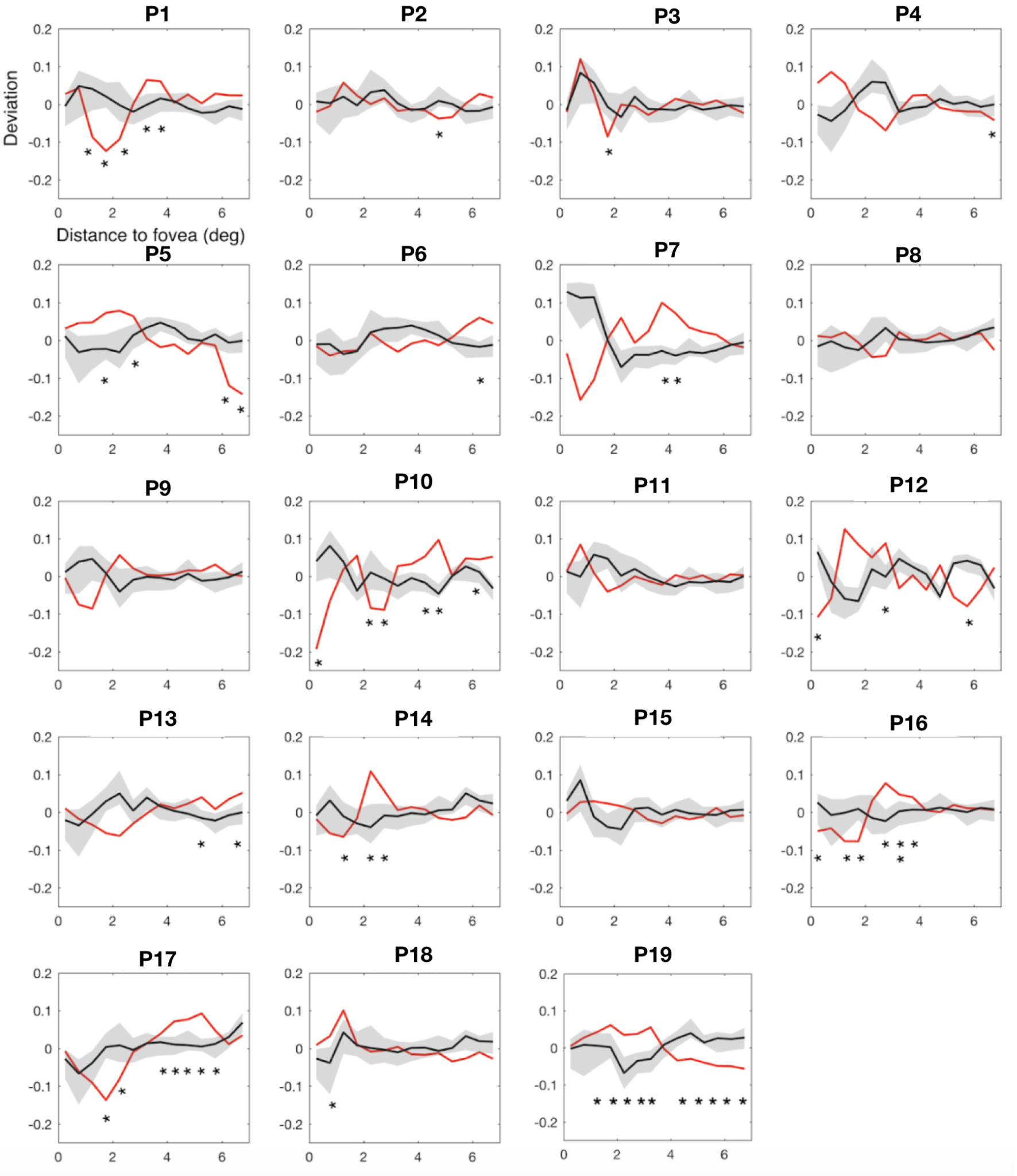


**Figure S8: V1 pRF eccentricity deviation for all participant pairs.** Deviation of the normalized number of voxels as a function of pRF eccentricity for control participants FF (black) and Glaucoma vs Control SS (red). The shaded gray area corresponds to 90% CI. The * indicate the bins for which the absolute deviation of the Glaucoma-Control SS pair was more extreme than any of control participants FF.


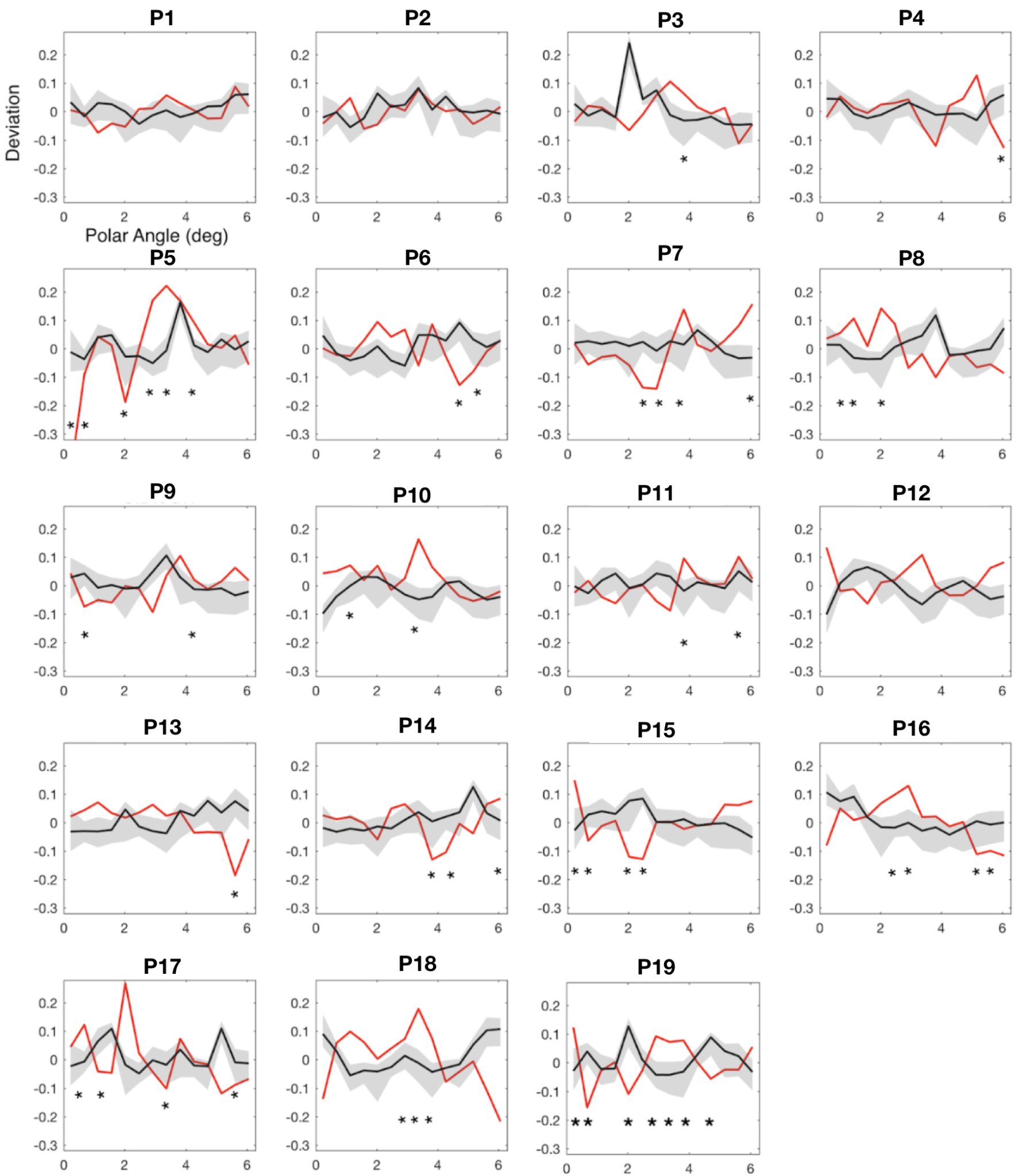


**Figure S9: V1 polar angle deviation for all participant pairs.** Deviation of the normalized number of voxels as a function of pRF size for control participants FF (black) and glaucoma participants vs control participant SS (red). The shaded grey are corresponds to 90% CI. The * indicate the bins for which the absolute deviation of the glaucoma-control SS participant pair was more extreme than any of control participants FF.

### **8. PRF position deviation as a function of contrast sensitivity and MNFL thickness**

To investigate how the local and individual differences in pRF position between participants with glaucoma and controls SS correlated with opthalmic measures at a gropup level, we ranked the deviation of the participant pair (either the glaucoma-control SS pair or the control SS-control NS pair) relative to the baseline deviation. The ranking was determined separately for each quarter of the visual field and for each bin of pRF properties (a total of 14 bins were used for eccentricity and polar angle). Per bin, the ranking was such that a participant pair that had a deviation that was larger than any of that of the controls (NS) was assigned a value of 19. A value of 1 was assigned in case a pair’s deviation was less than any of the controls. Intermediate values were assigned based on the ranking relative to the control (null) distribution. Next, per visual field quarter the ranks for all pairs were summed across bins. To derive a single pRF deviation measure the eccentricity and polar angle deviations were considered. This pRF deviation score per quarter of the visual field could vary between 14 (indicating the ranks of the pair in all eccentricity and polar angle bins were 1) and 266 (in case the pair would maximally differ from the control distribution in all 14 bins). Intermediate values imply a mix of ranks in different bins. The pRF deviation score obtained per quarter field was then correlated with the SAP scores (HFA and OCT) calculated per quarter field using a linear mixed effects model with a slope and intercept per subject as a random effect. Figure S10 shows that the pRF position deviations between participants with glaucoma and control participants SS correlated significantly with contrast sensitivity (r^2^=-0.24; p=0.004, panel A) but not with macular thickness (r^2^=-0.04; p=0.74, panel C). The deviation between control SS and NS does not correlate significantly with the simulated loss of contrast sensitivity (r^2^=-0.1; p=0.39, panel B).


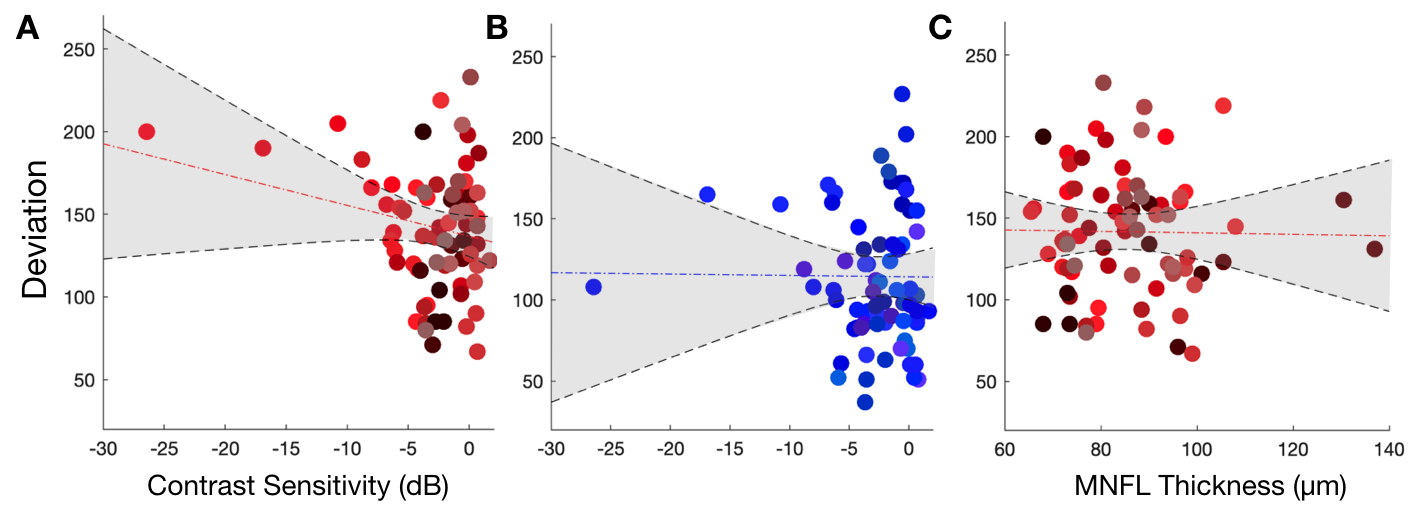


**Figure S10: V1 pRF position properties differ between participants with glaucoma and control participants. A, B and C**: Correlation between the deviation in pRF position properties and the contrast sensitivity ( A: glaucoma participants, B: Controls) and MNFL (glaucoma). Data are plotted per VF quadrant. For A and C, the deviation corresponds to the sum of the maximum rank of how much participants with glaucoma differ from their respective control SS compared to how much this specific control NS differs from all other control participants NS. Different colors denote the different participants. The dashed red line depicts the linear fit and the black line and shaded area corresponds to the 95% CI. For B the deviation corresponds to the sum of the maximum rank of how much control participants in SS condition differ from the NS condition compared to how much this specific control NS differs from all other control participants NS

### **9. Analysis of individual deviations in pRF properties based of the No Scotoma condition**

The use of simulated scotoma (SS) might come with pRF biases due to inaccurate SAP measurements or pRF modeling biases that are associated with the use of SS, and thus exacerbate the differences between glaucoma and control participants. To address the question of whether the differences measured between glaucoma and control participants are driven by our use of SS, we calculated the local deviants of pRF properties between participants with glaucoma and controls without a simulation (NS; Figure S11). In other words, we redid the analysis described in section 8 but now calculated the deviation between Glaucoma and controls NS. Similar to the results based on Controls SS, in all Glaucoma patients we see local variations in at least one of the pRF characteristics (size, eccentricity and polar angle; Figures S12, S13 and S14, respectively). However, when we aggregate the differences across participants (Figure S15), the total deviation vs contrast sensitivity relationship is no longer significant.


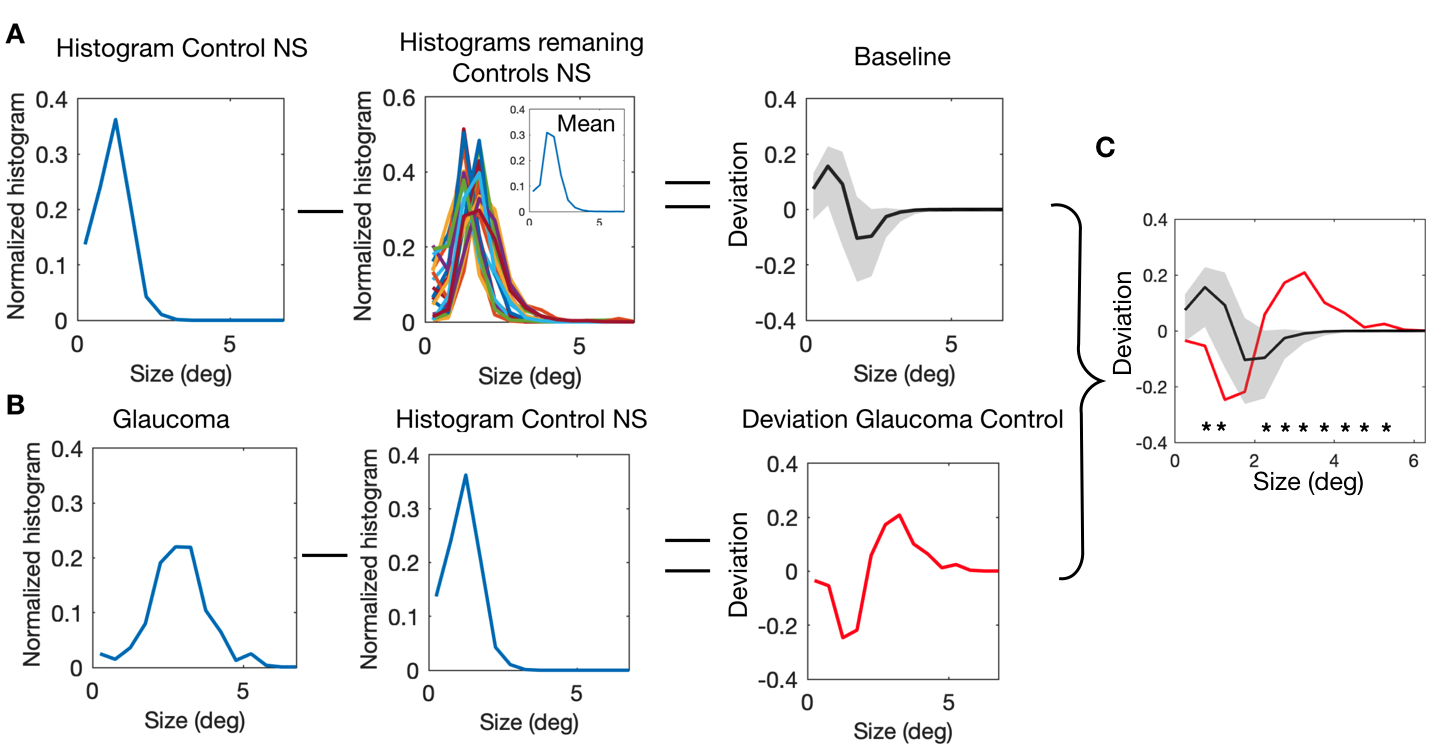


**Figure S11.** Similar scheme to Figure S6, however here the deviation is calculated between the histogram of the Glaucoma participant and the Control NS.


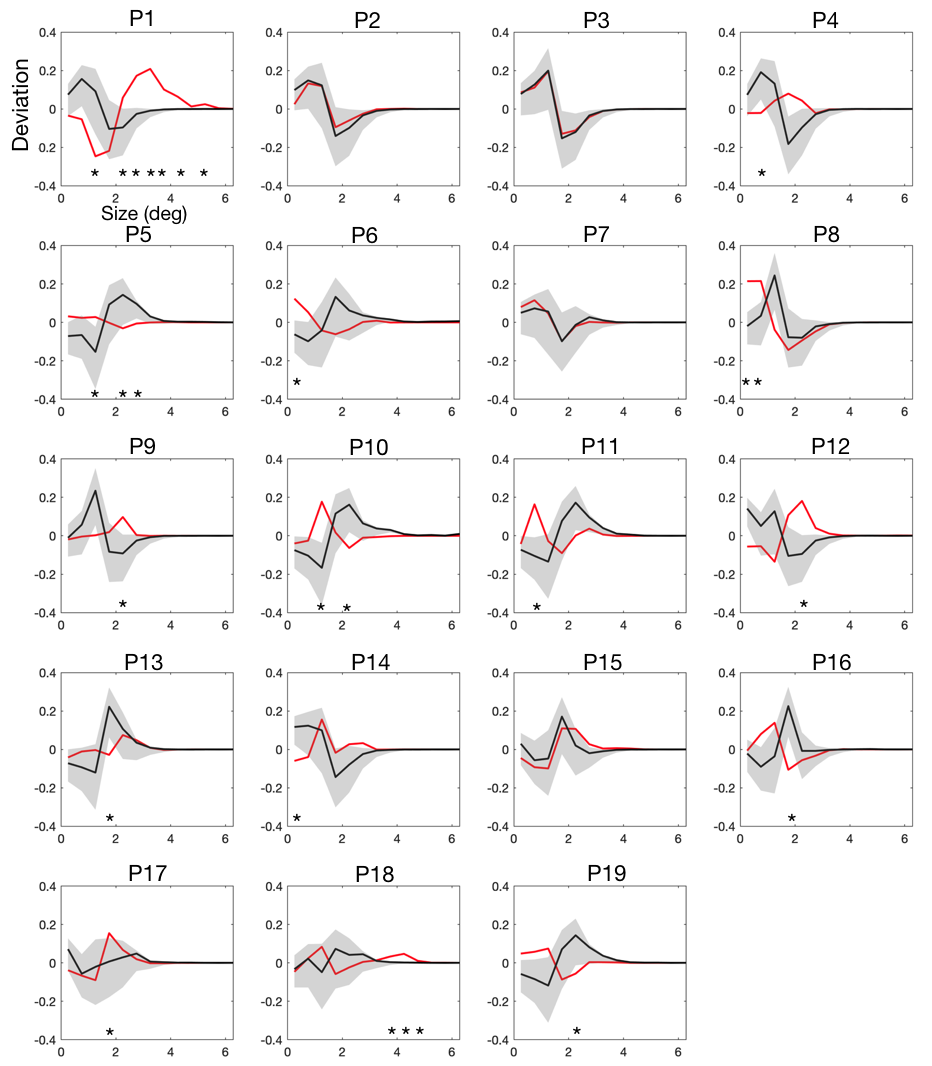


**Figure S12: V1 pRF size deviations (Glaucoma vs Control NS) for all participant pairs.** The graphs are similar to those of Figure S7.


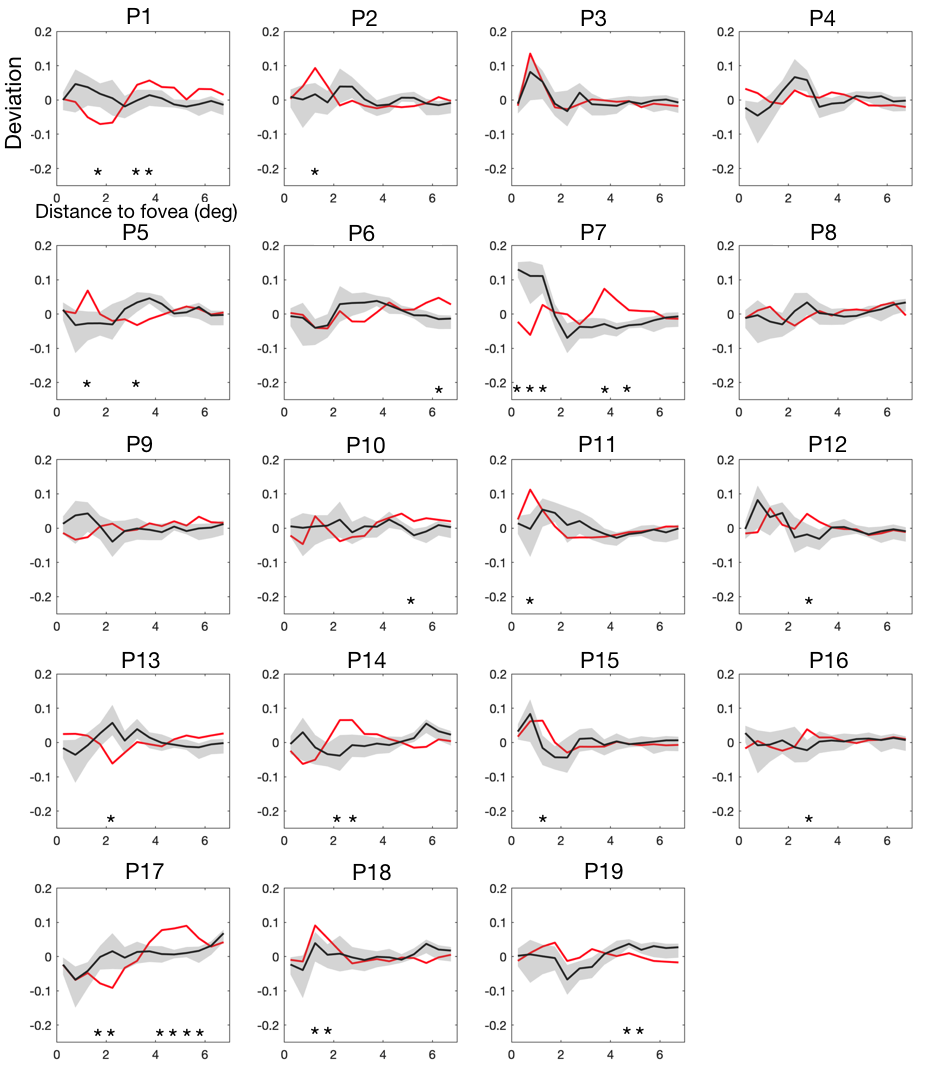


**Figure S13: V1 pRF eccentricity deviation (Glaucoma vs Control NS) for all participant pairs.** The graphs are similar to those of Figure S8.


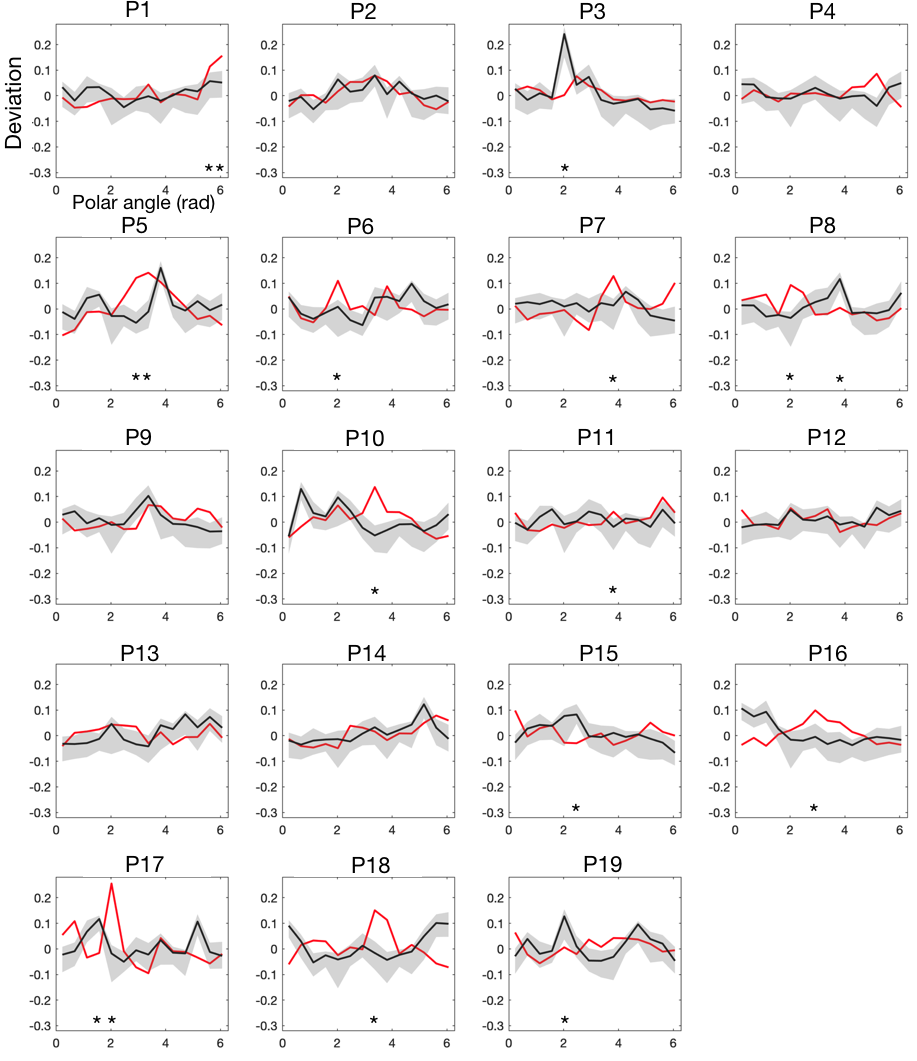


**Figure S14: V1 polar angle deviation (Glaucoma vs Control NS) for all participant pairs.** The graphs are similar to those of Figure S9.


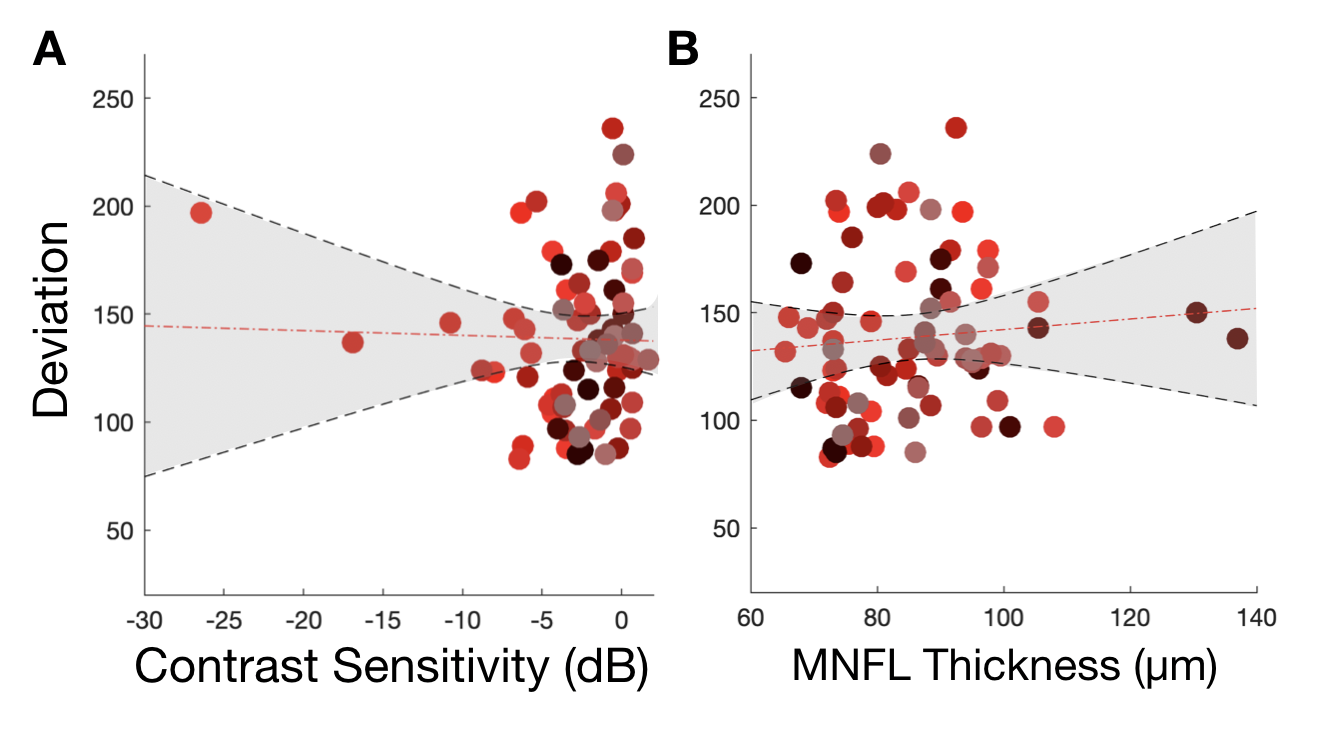


**Figure S15: V1 pRF position properties differ between participants with glaucoma and control participants NS.**  A and B: Correlation between the deviation in pRF position properties and the contrast sensitivity and MNFL, respectively. Data are plotted per VF quadrant. The deviation corresponds to the sum of the maximum rank of how much participants with glaucoma differ from their respective control NS compared to how much this control NS differs from all other control participants NS. Different colors denote the different participants. The dashed red line depicts the linear fit and the black line and shaded area corresponds to the 95% CI.

### Relation between Variance Explained and Contrast Sensitivity


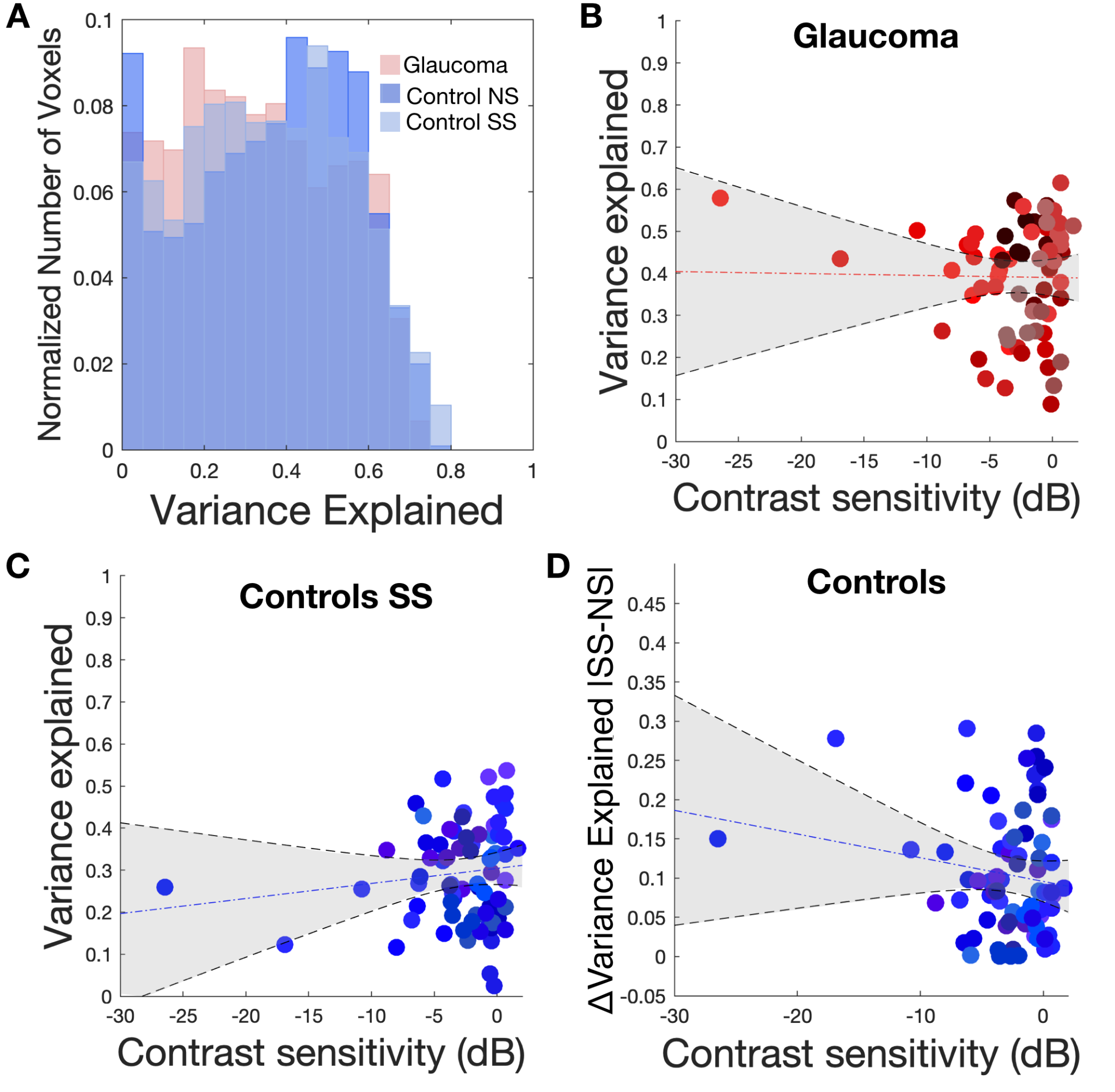


**Figure S16. Relation between pRF variance explained and the contrast sensitivity.** A: Histogram of the variance explained obtained for Glaucoma, Controls Ns and Controls SS. B and C: Correlation between the contrast sensitivity (dB) and the variance explained averaged per VF quadrant obtained for Glaucoma and Controls SS, respectively. D Module of variation of the VE averaged per VF quadrant between Controls SS and Controls NS.
